# Suture needles in Oral Surgery: Alterations depending on the type and number of sutures

**DOI:** 10.4317/medoral.17266

**Published:** 2011-12-06

**Authors:** Daniel Torres-Lagares, Sebastian Barranco-Piedra, Angela Rodríguez-Caballero, María-Angeles Serrera-Figallo, Juan-José Segura-Egea, José-Luis Gutiérrez-Pérez

**Affiliations:** 1 Prof. Dr. Oral Surgery. Universidad de Sevilla. Seville, Spain; 2 Prof. Master in Oral Surgery. Universidad de Sevilla. Seville, Spain; 3 Master in Oral Surgery. Universidad de Sevilla. Seville, Spain; 4 Prof. Dr. Dentistry in handicapped Patients. Universidad de Sevilla. Seville, Spain; 5 Prof. Dr. Conservative Dentistry. Universidad de Sevilla. Seville, Spain; 6 Head of Oral and Maxillofacial Surgery Department (on extended leave). Hospital Universitario Virgen del Rocío. Seville, Spain

## Abstract

This study examined whether the number and type of sutures used in oral surgery influence two ad hoc variables (incision plane and displaced area), which are two variables related to whether the suture needle is suitable for the task. Seventy-five TB-15 needles were studied, which were used to suture between zero and three mucosa and/subperiosteal sutures, producing 15 groups with 5 needles in each one. The incision plane and displaced area were measured for each group, which are two variables related to how the needle has worn and altered. Statistical treatment was conducted using the Kruskal-Wallis H test to compare multiple values and the Mann-Whitney U test to compare pairs. A multi-stage regression model was applied with the aim of predicting the changes in the dependent variables based on the number and type of sutures performed. The incision plane ranged from 126.67 to 346.24μm among the different groups. The displaced area was measured as being between 14 524.83μm² and 128 311.91μm². The best predictive model for the incision plane obtained a coefficient of determination (R2) of 0.149, while it reached 0.249 for the displaced area. Subperiosteal sutures held more weighting among the variables studied. Mucosal sutures did not seem to greatly affect needle wear. Observations reported in this paper indicate that the needle should be changed after having performed two subperiosteal sutures, given the wear and change to the incision plane that is produced, which causes the needle’s cutting ability to reduce.

** Key words:** Needles, sutures, material testing, oral surgery, third molar, scanning electron microscope.

## Introduction

Soft-tissue wound healing is a complex reparative process that leads to tissue regeneration and restitutio ad integrum, or, failing that, injured tissue is replaced with a fibrous scar tissue (1).

Wound treatment (either after an accident or surgery) is effective as it does not interfere with the natural healing process and helps in its successive stages (2). Correct approximation of the wounds’ edges makes primary closure easier, therefore benefiting the healing process (3). As such, the suture and its correct use play a crucial role in this process. 

Understanding the biological interaction between the suture material and the tissue to be sutured is essential to ensure optimum tissue handling (2,4). Currently, needles are mostly manufactured using surgical stainless steel (AISI 420) (5,6) and silk is used for the thread (3,7). Steel is the perfect material for this function due to its toughness, biocompatibility, corrosion resistance, and because it is easy to machine (3). Tissue inflammation upon contact with the thread and intrinsic trauma caused by needle insertion, are the main suture-tissue interactions (4,8,9).

Two solid surfaces in relative motion come in contact with one another during the suturing process: the needle and the tissue to be sutured. This causes both surfaces to wear as the two materials in contact progressively erode on a microscopic level (10,11). 

A worn needle can hinder the intervention as it does not penetrate the tissue easily, can tear the tissue, make it difficult to correctly position sutures and make the intervention longer (1-3). 

There is currently a wide range of needles specifically designed for different types of tissues, aimed to cause as little damage as possible (8,12). Several studies have been conducted with regards the surgical intervention and its relationship with healing (13), however, after having revised the literature published to date, not a single article examining how suture needles alter with use was found.

The objective of this study is to assess the wear to needles used in oral surgery, depending on the number and type of sutures. It also aims to predict at what point the needle should be changed, so as to be able to continue suturing optimally. 

## Material and Methods

This experimental, prospective and controlled study was conducted in the Faculty of Odontology in Seville from January to June 2009. The independent variables were the number and type of sutures performed, examining how they influenced needle wear. 

Seventy-five 20mm-TB-15 needles were included (3/8th circle reverse cutting) (Lorca Marín, Spain). All needles were used to suture bayonet flap incisions after removing impacted lower right molars. 

Needles were arranged into different groups according to the type of suture (subperiosteal or mucosa) and the number of sutures (0 to 3) performed, with 5 needles in each study group.

The following were excluded: needles used to perform interdental sutures, sutures for brace and dental steel band wearers, or those that did not comply with the previously mentioned characteristics. 

One surgeon performed all of the sutures so as to prevent intraoperator bias and achieve a homogenous sample, where the suture conditions for each needle were as similar as possible. 

A bayonet incision was performed to remove impacted third molars. So that the study was homogeneous, all incisions were performed until the mesial contact of the second molar, vertically and extending 1cm distally. Simple sutures were used: the entry and exit sutures had to be at the same distance from the wound edges (between 3 and 5mm). 

We used two types of sutures: mucosal sutures - the needle did not have any contact with hard tissue during the process; and subperiosteal sutures - the needle came into contact with the bone crest. Mucosal sutures were performed to close the distal incisions and subperiosteal sutures for vertical incisions. Together with the type of suture, the number of sutures performed were considered and classified as a study group. The needles were collected, but were not washed or sterilised so as not to cause any other type of alteration, therefore maximum care was taken when handling them.

A scanning electron microscope (SEM) was used to observe the wear produced. Needles from the same group were placed onto a microscope slide and sputter-coated with gold to aid electron emission, making it easier to visualise them using the SEM. The microscope slide was then placed in the SEM vacuum chamber and each of the needles was observed and photographed. 

**Figure 1:**
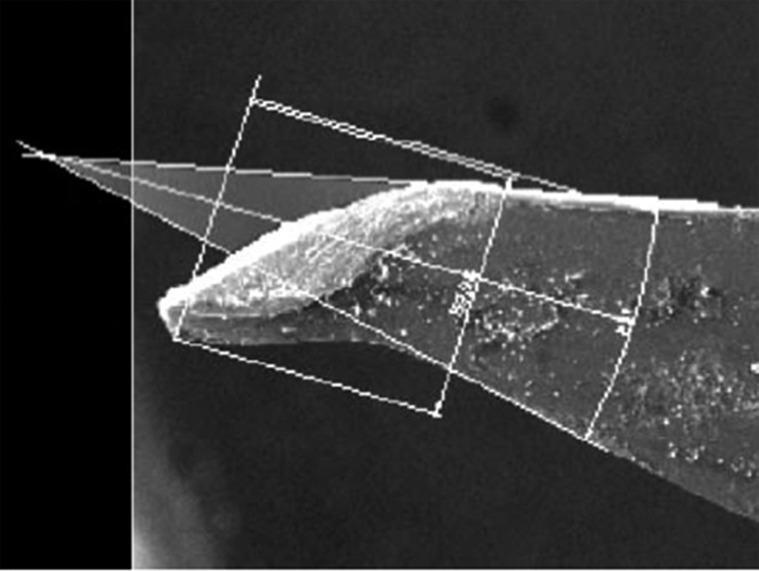
Measuring the displaced area and incision plane on a study needle.

**Figure 2:**
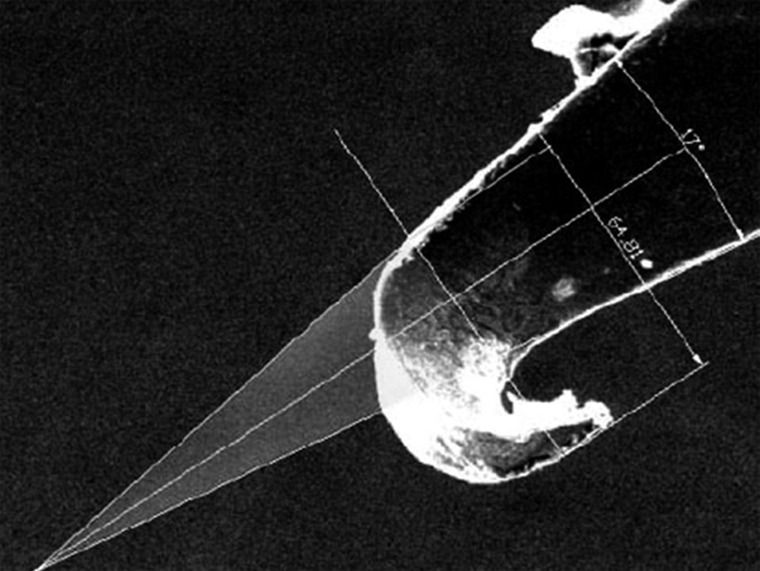
Measuring the displaced area and incision plane on a study needle.

Two ad hoc variables, incision plane and displaced area, were then assessed. To do so, two lines were traced along the lateral edges of the needles until they met at an imaginary point. The empty area between the point of the needle and the imaginary point between these two lines was the displaced area (Figs. 1,2). 

The incision plane is the width of the needle point that goes through the tissue so that the unaltered needle edges continue suturing. It was measured by using a straight line from the outermost part of the damaged needle edge to the intact needle edge (Figs. 1,2).

The descriptive and inferential statistical data analysis was performed using SPSS v.11 statistical software for Windows. The Kruskal-Wallis H test was used to compare multiple values and the Mann-Whitney U test to compare pairs.

A multi-stage regression model was applied with the aim of predicting the changes between the dependent variables in view of the number and type of sutures performed.

## Results

The values obtained from measuring the incision plane and displaced area for the relevant study groups are presented in  Table 1 A.The incision plane ranged from 126.67 to 346.24µm among the different groups. The displaced area ranged between 14 524.83µm² and 128 311.91µm². 

Both the largest incision plane and displaced area were found in the three-subperiosteal suture group (346.24µm±29.24 and 128 311.91µm2±39 406.75, respectively). 

 Table 1 B shows the results of the variables incision plane and displaced area for the same number of mucosa and subperiosteal sutures. The greater the number of subperiosteal sutures, the larger the values for incision plane and displaced area (from 130.07µm±89.67 to 227.75µm±82.51 and from 15 207.32µm2±12 182.03 to 46 252.97µm2±52 323.41). However, this correlation did not occur with the number of mucosal sutures performed. 

 Table 2 shows the different models studied to predict the variations in the two variables based on the number of mucosal sutures, number of subperiosteal sutures, a combination, the data which identifies these models and their goodness-of-fit. 

The best predictive model for incision plane obtained a R2 of 0.149, while it reached 0.249 for the displaced area.

**Table 1:**
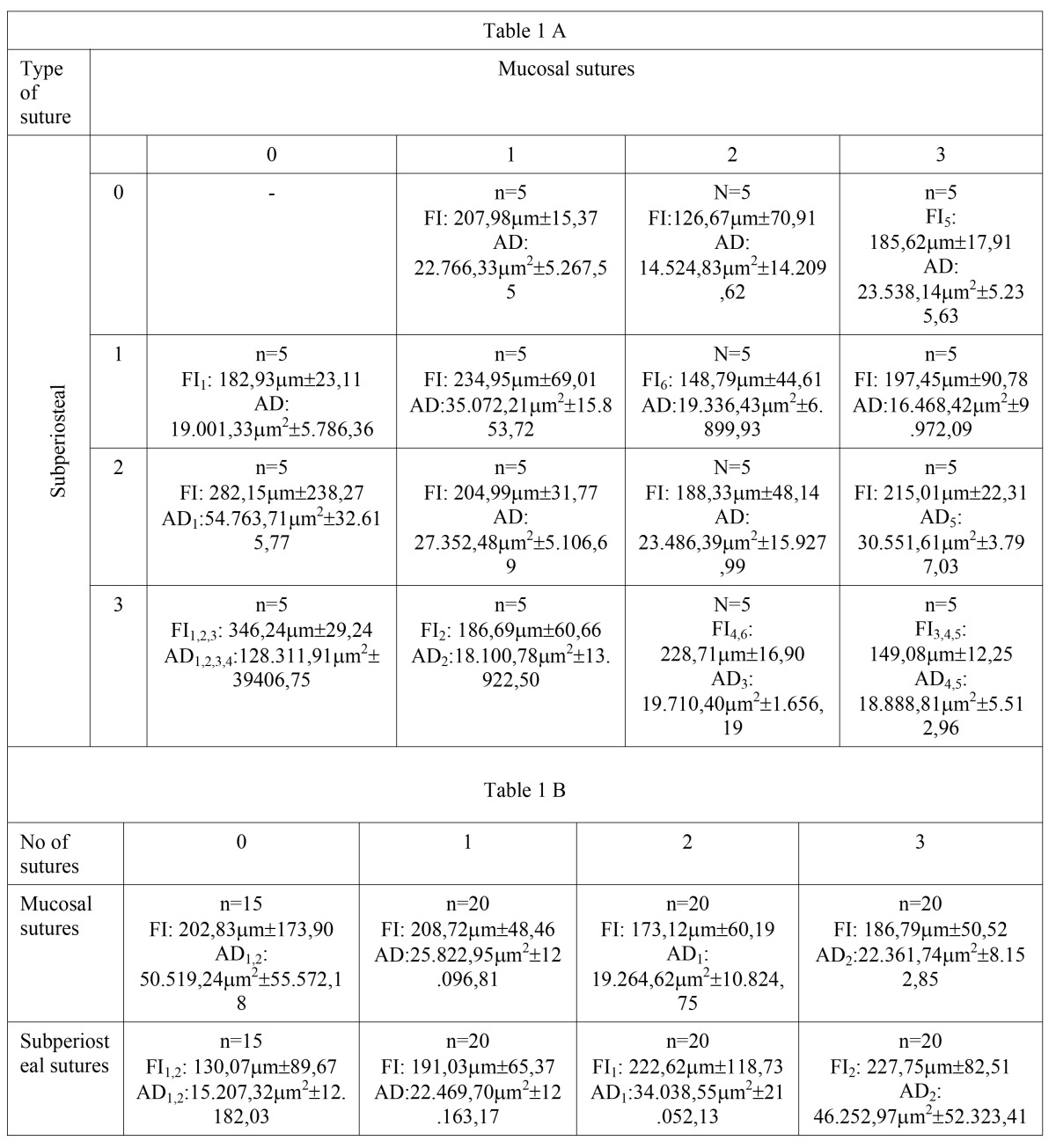
1.A) – Number of needles per study group, incision plane (FI) and displaced area (AD). Statistically significant differences (P<0.05) are indicated per sub-index pair (1-1, 2-2, etc.). Table 1.B) - Number of needles per study group, incision plane (FI) and displaced area (AD) per group of needles according to the number of mucosa or subperiosteal sutures performed. Statistically significant differences (P<0.05) are indicated per sub-index pair (1-1, 2-2, etc.)..

## Discussion

Examining the alterations produced to suture needles is completely justified when considering the influence that they may have on wound closure and the healing processes. 

We did not find any articles examining the alterations produced to the suture needle point through use in the literature review (13). However, we did find studies describing new designs, sizes, categories, and indications of the different types of needles (1,2), as well as one article about a needle that was lost in the tonsillar area during surgery (12).

The main research limitation of our study was that the values were given in two-dimensional measurements, where in reality they should be three-dimensional. This aspect, as well as the innovative variables that were used, meant that results had to be interpreted with caution, although we believe that they are useful. 

It is necessary to highlight that incision plane did not change significantly the more mucosal sutures were used ( Table 1.B). However, there was statistical significance for the incision plane when using subperiosteal sutures. It was significantly greater in the two and three subperiosteal sutures groups compared with those that did not have any subperiosteal sutures. If we were to apply these results to practice, a needle should be changed, at least, after performing two subperiosteal sutures.

This point was confirmed with the data in  Table 1.A, where three of the statistically significant differences are found in the same column, i.e. groups which differ in the number of subperiosteal sutures. 

Only two statistically significant differences were found in the same row (in the three-subperiosteal suture group, between the groups did not use needles for mucosal sutures and those that did). Using more mucosal sutures may have caused the needle point to become softer and the incision plane slightly reduced in these last groups, according to the data obtained.

The displaced area altered when considering both the number of mucosal sutures and subperiosteal sutures performed. 

The greater the number of subperiosteal sutures used, the larger the displaced area (significant difference between the group without subperiosteal sutures and the groups with two or three subperiosteal sutures). This point should be considered when deciding if the needle needs to be changed.

However, it was the opposite for mucosal sutures, which implies that they are not relevant in healing. 

So as to provide more details on the behaviour described above,  Table 1.A clearly shows that the three subperiosteal sutures and zero mucosal suture groups present an unusually large displaced area. This determined that, of the five statistically significant relationships found for this variable on this table, three are in the same row, among this group and the other three in the same row. 

Logically, we cannot assume that when more sutures are performed, needle wear reduces, which would mean that the contact with the tissue causes material to add to the needle. This therefore shows us that the way that subperiosteal sutures are performed holds great importance to the study variables. 

It was only possible to predict the alterations (incision plane and displaced area) according to the type and number of sutures for some of the variables studied. 

Being consistent with the analysis and discussion of the results, the incision plane was predicted suitably depending on the number of subperiosteal sutures performed (P<0.001; R2=0.129). The prediction was unsuitable (P=0.394, R2=0.009) if only the number of mucosal sutures performed was used. When both variables were used, the variable related to mucosal sutures remained insignificant (P=0.36), despite the adjustment having increased slightly (R2=0.149).

From these data, it seems that it would be more logical to use the number of subperiosteal sutures to predict an increase in the incision plane.

Both the individual use of the number of mucosal sutures (R2=0.107; P=0.003) and subperiosteal sutures (R2=0.142; P=0.001) were of importance to predicting the displaced area. Using both of them in a predictive model notably improved the coefficient of determination (0.249 compared with 0.142 or 0.107), with a stable predictive value for both data. 

Nevertheless, there is still room for improving the adjustment of a hypothetical model that predicts how the needle use variables to be studied may change. If we were to increase the number of needles studied, and therefore increase the sample size, perhaps it would provide us with more reliable and better adjusted prediction models. New variables should also be assessed, such as suturing time, instruments handled, surgeon’s experience, etc. Meanwhile, our models should be empirically tested when predicting alterations in needles and sutures.

Examining and controlling variables such as incision plane and displaced area is fundamental, given that alterations at this level reduce the needles’ ability to penetrate and cut, meaning that the surgeon has to make a greater effort to ensure that the needle goes through the tissue. We believe that the definition of these variables is suitable for our study, which revealed that subperiosteal sutures have a greater impact on them than mucosal sutures.

This importance is supported by the great weighting that this aspect holds in all the prediction models. Therefore, based on our data, it seems logical to recommend that the suture needle is changed after performing two subperiosteal sutures, although this recommendation should be taken with caution.

Lastly, we can highlight two more limitations in our study. On one hand, it is impossible to assess the control variable surgeon quality or ability (always using the same surgeon, using standard material, and not using magnifiers). However, it is logical and we are certain that solid surgical training in advanced tissue and suturing techniques (including microsurgery techniques) would significantly influence the results obtained. Therefore, our data may only be extrapolated to similar experiences, training and technologies as those used in our study.

On the other hand, to achieve homogeneity in the study we chose to only observe the wear produced from bayonet incisions. Nevertheless, there are many types of incisions in oral surgery and each one has its own anatomical and performance determining factors. It would be interesting to conduct future studies that examine how the type of incision and intraoral location, or the number of mucosa or subperiosteal sutures may influence needle alterations.

We can reach the following conclusions from our results:

1. Subperiosteal sutures, as have been defined, had more importance among the variables studied (displaced area and incision plane). Mucosal sutures did not seem to greatly affect needle wear.

2. The needle should be changed after having performed two subperiosteal sutures, given that it shows an important change in wear and incision plane, which causes the needle’s cutting ability to reduce.

**Table 2:**
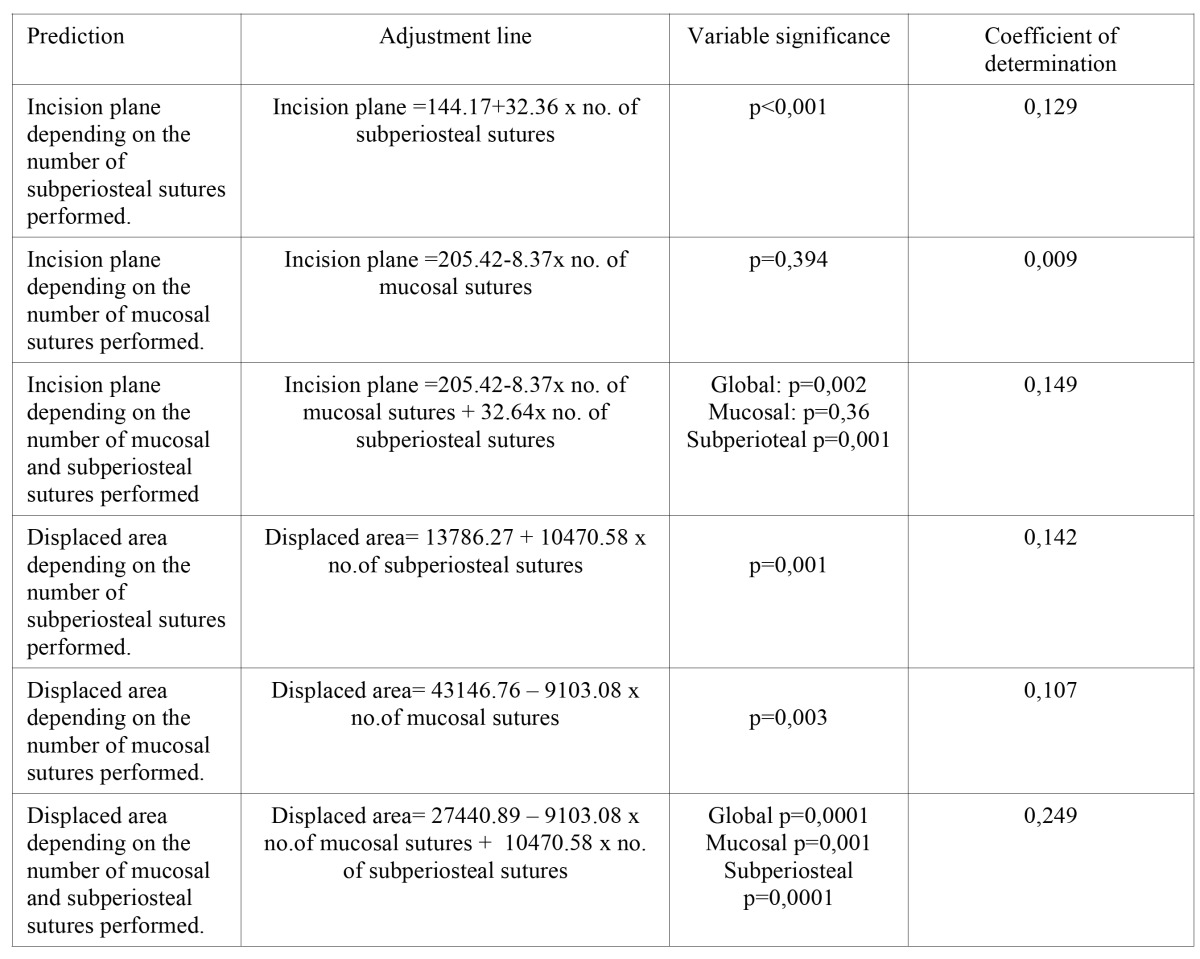
Adjustment model to predict the variables incision plane and displaced area depending on the number of mucosa and subperiosteal sutures performed.
